# Comparison of Perioperative, Functional, and Oncologic Outcomes of Open vs. Robot-Assisted Off-Clamp Partial Nephrectomy: A Propensity Scored Match Analysis

**DOI:** 10.3390/s24092822

**Published:** 2024-04-28

**Authors:** Riccardo Mastroianni, Giuseppe Chiacchio, Leonard Perpepaj, Gabriele Tuderti, Aldo Brassetti, Umberto Anceschi, Mariaconsiglia Ferriero, Leonardo Misuraca, Simone D’Annunzio, Alfredo Maria Bove, Salvatore Guaglianone, Rocco Simone Flammia, Flavia Proietti, Marco Pula, Giulio Milanese, Costantino Leonardo, Andrea Benedetto Galosi, Giuseppe Simone

**Affiliations:** 1 Urology, IRCCS “Regina Elena” National Cancer Institute, 00128 Rome, Italy; riccardo.mastroianni@ifo.it (R.M.); gabriele.tuderti@ifo.it (G.T.); aldo.brassetti@ifo.it (A.B.); umberto.anceschi@ifo.it (U.A.); maria.ferriero@ifo.it (M.F.); leonardo.misuraca@ifo.it (L.M.); simone.dannunzio@ifo.it (S.D.); alfredo.bove@ifo.it (A.M.B.); salvatore.guaglianone@ifo.it (S.G.); roccosimone92@gmail.com (R.S.F.); flavia.proietti@ifo.it (F.P.); marco.pula@ifo.it (M.P.); costantino.leonardo@ifo.it (C.L.); puldet@gmail.com (G.S.); 2 Urology Division, Azienda Ospedaliero-Universitaria delle Marche, Università Politecnica delle Marche, 60126 Ancona, Italy; leonardperpepaj@gmail.com (L.P.); g.milano972@gmail.com (G.M.); galosiab@yahoo.it (A.B.G.)

**Keywords:** partial nephrectomy, off-clamp, renal function, renal cancer, robot, open surgery

## Abstract

Off-clamp partial nephrectomy represents one of the latest developments in nephron-sparing surgery, with the goal of preserving renal function and reducing ischemia time. The aim of this study was to evaluate and compare the functional, oncologic, and perioperative outcomes between off-clamp robot-assisted partial nephrectomy (off-C RAPN) and off-clamp open partial nephrectomy (off-C OPN) through a propensity score-matched (PSM) analysis. A 1:1 PSM analysis was used to balance variables potentially affecting postoperative outcomes. To report surgical quality, 1 year trifecta was used. Univariable Cox regression analysis was performed to identify predictors of trifecta achievement. The Kaplan–Meier method was used to compare cancer-specific survival (CSS), overall survival (OS), disease-free survival (DFS), and metastasis-free survival (MFS) probabilities between groups. Overall, 542 patients were included. After PSM analysis, two homogeneous cohorts of 147 patients were obtained. The off-C RAPN cohort experienced shorter length of stay (LoS) (3.4 days vs. 5.4 days; *p* < 0.001), increased likelihoods of achieving 1 year trifecta (89.8% vs. 80.3%; *p* = 0.03), lower postoperative Clavien–Dindo ≤ 2 complications (1.3% vs. 18.3%, *p* < 0.001), and lower postoperative transfusion rates (3.4% vs. 12.2%, *p* = 0.008). At univariable analysis, the surgical approach (off-C RAPN vs. off-C OPN, OR 2.22, 95% CI 1.09–4.46, *p* = 0.02) was the only predictor of 1 year trifecta achievement. At Kaplan–Meier analysis, no differences were observed between the two groups in terms of OS (log-rank *p* = 0.451), CSS (log-rank *p* = 0.476), DFS (log-rank *p* = 0.678), and MFS (log-rank *p* = 0.226). Comparing RAPN and OPN in a purely off-clamp scenario, the minimally invasive approach proved to be a feasible and safe surgical approach, with a significantly lower LoS and minor rate of postoperative complications and transfusions as a result of improved surgical quality expressed by higher 1 year trifecta achievement.

## 1. Introduction

Renal cell carcinoma (RCC) stands as one of the prevalent urological malignancies, posing a significant healthcare challenge globally. This aggressive cancer exhibits geographic variations, with a higher burden observed in men than women in developed nations, with an estimated male-to-female ratio of 1.5:1.0. The peak incidence typically occurs between 60 and 70 years of age, and established risk factors associated with RCC development include tobacco smoking, hypertension, and obesity. Renal cell carcinoma comprises a heterogeneous group of cancers with different genetic and molecular features reflecting the different histological subtypes, each of which is characterized by unique alterations at the cellular level. Clear cell, papillary (types 1 and 2), and chromophobe RCC constitute the most common solid renal malignancies, collectively accounting for approximately 85–90% of all diagnosed kidney cancers. Despite a rising overall incidence, recent decades have witnessed encouraging improvements in relative survival rates for RCC patients [1]. This appears to be due to increasingly earlier diagnoses and technological advances in robotic renal surgery that have led to increasingly safe and efficient surgeries.

The evolution of preoperative staging and surgical techniques over the past decades has positioned partial nephrectomy (PN) as the gold standard for localized kidney cancer [2], providing improved renal function preservation and comparable oncological outcomes to radical nephrectomy [3,4]. Consequently, indications of nephron-sparing surgeries have been increased, supporting PN whenever technically feasible [5,6].

The optimal PN should achieve negative surgical margins and minimize postoperative complications and renal function impairment [7]. The modifiable factors influencing postoperative renal function include the enucleoresection technique [8], the renorrhaphy technique [9], and the duration of renal ischemia.

The impact of renal ischemia on renal function remains a matter of debate [10,11]. While the earlier literature emphasized the critical role of ischemia time [11], recent findings from a multicenter randomized control trial reported no significant differences in long-term functional outcomes comparing on- vs. off-clamp PN [12]. However, PN techniques have evolved with the goal of reducing ischemia damage, not only through the reduction of clamping duration but also through the implementation of selective clamping or the omission of any arterial clamping [13,14].

PN can be performed through laparoscopic, robotic, or open approaches [15,16,17]. European guidelines pointed out that the choice of the approach is secondary, emphasizing the importance of performing nephron-sparing surgery (NSS) whenever technically feasible, regardless of the type of surgical approach [7].

Previous studies comparing robot-assisted PN (RAPN) with open PN (OPN) have generally favored RAPN, particularly in terms of complication rates, estimated blood loss, and length of stay (LoS) [18,19,20]. However, the comparison in a strictly off-clamp setting is still underinvestigated [20]. In this off-clamp scenario, the application of the robotic technique in association with the most recent technologies in the field of preoperative and intraoperative planning, such as artificial intelligence-based imaging processing, preoperative 3-D model, or intraoperative augmented reality and elastography, could lead to an increasingly effective and less invasive NSS surgery, helping to choose the right surgical approach for the right patient. This implementation of different technologies was made possible by the robotic platform, which can integrate, apply, and collect feedback during the operative time.

The aim of this study was to compare functional, oncologic, and perioperative outcomes of off-clamp RAPN (off-C RAPN) vs. off-clamp OPN (off-C OPN) with a propensity score-matched (PSM) analysis.

## 2. Methods

### 2.1. Patients and Dataset

Data were prospectively gathered from an institutional review board-approved database encompassing two institutes queried for patients who underwent PN for unifocal renal tumors (cT1-2) from January 2012 to December 2022. Inclusion criteria were TC or RM detection of the renal neoformation, unifocality of the neoformation, cT ≤ 2, cN0, cM0, any R.E.N.A.L. score, and open or robotic surgical approaches. Exclusion criteria were the presence of solitary kidney, hematuria, laparoscopic surgery, and not localized or metastatic tumor.

The surgical approach was selected individually by surgeons. Both surgeons were experienced in PN, with an average of at least 50 PN per year over the past 3 years. All patients underwent off-clamp PN with the enucleation technique. The sliding-clip technique of renorrhaphy was performed in all patients [21]. All robot-assisted NPs were performed in the same center, while open NPs were performed in both centers.

Baseline characteristics, including age, sex, BMI, comorbidities, smoking status, preoperative hemoglobin (HB), preoperative estimated glomerular filtration rate (eGFR), chronic kidney disease (CKD) stage, and American Society of Anesthesiologists (ASA) score, were systematically recorded. Tumor characteristics were collected, and surgical complexity was described according to R.E.N.A.L. score [22], categorizing tumors into low (R.E.N.A.L. score 4–6), moderate (R.E.N.A.L. score 7–9), or high (R.E.N.A.L. score 10–12) risk groups.

Renal function was evaluated using serum creatinine and eGFR, calculated using the Modification of Diet in Renal Disease (MDRD) formula [23]. As stated by the National Kidney Foundation (NKF) guidelines, a postoperative eGFR decrease of over 30% was classified as “significant renal function deterioration” (sRFD) [24].

Intra- and postoperative complications were defined according to the Clavien–Dindo (CD) classification system [25]. Major complications were defined by CD ≥ 3.

Surgical quality was assessed using 1 year trifecta, previously described and defined as negative surgical margins, absence of CD ≥ 3 complications, and eGFR reduction < 30% [26].

Intraoperative and 12 h postoperative fluid management by the anesthesiologist was based on cardiac output using a Vigileo-FloTrac system [27].

Complications and adverse events were recorded during the inpatient stay, on readmission, and in outpatient clinics. Research nurses collected and independently submitted outcome data.

### 2.2. Statistical Analysis

Continuous variables were reported using mean and standard deviation (SD) and compared using the Student *t*-test. Categorical variables were described using frequencies and proportions, and the comparison was performed using the chi-square test. Due to inherent disparities between cohorts, we performed a 1:1 PSM analysis with a caliper of 0.3 to account for these differences. Employing the propensity score method helped mitigate the common biases associated with conventional multivariable modeling. We adjusted for age, R.E.N.A.L. nephrometry score, tumor size, and preoperative HB as variables (*PSM calculation formula: set.seed(11) matchit (approach ~ Renal + Age + size + preop_HB, data = dat, method = “nearest”, replace = F, ratio = 1, caliper = 0.3)- > p*). Univariable Cox regression analysis was used to identify predictors of trifecta achievement. The Kaplan–Meier method was performed to assess survival outcomes, described as cancer-specific survival (CSS), overall survival (OS), disease-free survival (DFS), and metastasis-free survival (MFS) probabilities.

The significance level was set at <0.05. Statistical analysis was conducted using the Statistical Package for the Social Sciences (SPSS v.21; IBM Corporation, Armonk, NY, USA), the R statistical software v. 4.3.2 (R Foundation for Statistical Computing; Vienna, Austria), and Stata software (v. 8; StataCorp LLC, Lakeway Drive College Station, TX, USA).

## 3. Results

Out of 542 patients included in the study, 395 underwent off-C RAPN and 147 off-C OPN.

Baseline, perioperative, and functional data distribution between the two groups are reported in Table 1. Particularly, patients who underwent RAPN were significantly younger (60.2 vs. 64.5, *p* < 0.001), while BMI (26.8 vs. 26.9, *p* = 0.87), diabetes rate (12.4% vs. 12.2%, *p* = 1), and hypertension rate (50.6% vs. 54.4%, *p* = 0.4) were comparable between the two groups, as well as ASA score, preoperative HB (14.5 vs. 13.6, *p* = 0.1), and preoperative eGFR (65.6 vs. 65.7, *p* = 0.9). However, a statistically significant difference was detected between cohorts in terms of tumor size (4.5 vs. 3.5, *p* < 0.001) and R.E.N.A.L. score (low risk: 26.1% vs. 46.9%; moderate risk: 45.8% vs. 42.9%; high risk: 28.1% vs. 10.2%; *p* < 0.001). No differences were found between the two groups regarding the preoperative CKD stage (*p* = 0.76).

After PSM analysis, two homogeneous cohorts, each of 147 patients, were obtained (Table 1).

**Figure 1:**
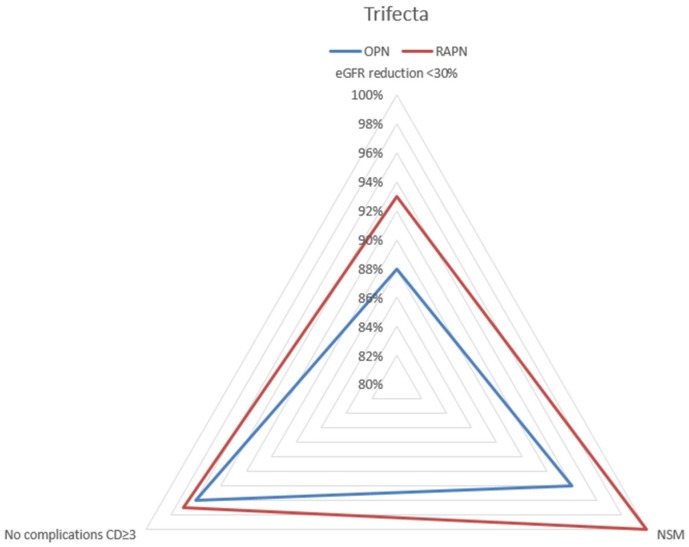
Trifecta achievement in off-C OPN group and off-C RAPN group.

Patients who underwent RAPN had shorter hospital stays (3.4 days vs. 5.4 days; *p* < 0.001) and an increased likelihood of achieving 1 year trifecta (89.8% vs. 80.3%; *p* = 0.03) (Figure 1).

On the one hand, no differences were detected in terms of intraoperative complications (8.8% vs. 10.2%, *p* = 0.7). On the other hand, a statistically significant difference was detected in terms of postoperative complication rates (3.4% vs. 21.7%, *p* < 0.001), predominantly related to higher low-grade (CD ≤ 2) complication rates that occurred in the OPN cohort (1.3% vs. 18.3%, *p* < 0.001), while postoperative complications CD > 3 rates were 2.1% and 3.4% in the RAPN and OPN groups, respectively. As a result, the OPN cohort experienced a higher rate of postoperative transfusions (3.4% vs. 21.7%, *p* = 0.008), while no differences were detected in terms of HB at discharge (13.1 vs. 11.7; *p* = 0.16) and intraoperative transfusion rates (0.7% vs. 3.4%; *p* = 0.21) (Table 2). Overall, no conversion to radical nephrectomy was required for both cohorts.

In terms of functional outcomes, no significant differences were observed for CKD stage migration ≥ 3a (10.9% vs. 14.2%; *p* = 0.48).

At the final anatomopathological exam, the distribution of the pT stage and histology was similar between the RPN and OPN groups. The majority of patients in both groups had pT stage 1a tumors (74.1% vs. 73.5%, *p* = 0.9). The proportion of patients with pT stage 1b and 2a tumors was comparable between the two groups too (23.1% vs. 23.8%, *p* 0.9 and 2.7% vs. 2.7%, *p* = 1, respectively).

Clear cell renal carcinoma was the most common tumor histology in both groups, with a slightly higher but not statistically significant prevalence in the RPN group (62.5% vs. 55.1%, *p* = 0.23). Papillary tumors of type 1 were 6.1% vs. 7.5% in the two groups (*p* = 0.81), while type 2 were 5.4% vs. 6.1% (*p* = 1). The OPN group had a slightly higher but not statistically significant proportion of benign tumors (32.0% vs. 25.8%, *p* = 0.3).

At univariable analysis, the surgical approach was the only predictor of 1 year trifecta achievement (off-C RAPN vs. off-C OPN, OR 2.22, 95% CI 1.09–4.46, *p* = 0.02) (Table 3).

However, the OPN cohort reported a higher rate of positive surgical margins (0% vs. 6.1%, *p* = 0.003).

**Figure 2:**
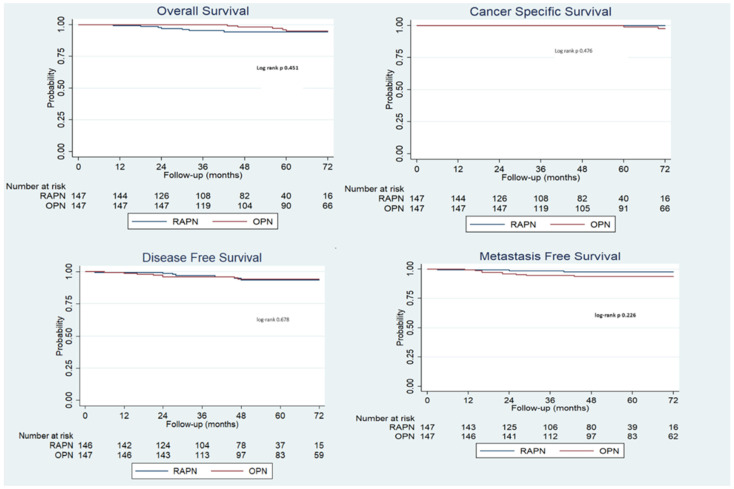
Kaplan–Meier of overall survival, cancer-specific survival, disease-free survival, and metastasis-free survival of the propensity score match population.

Finally, an analysis of survival outcomes was performed. At Kaplan–Meier analysis, at a median follow-up of 64 (IQR 49; 75), no statistically significant differences were detected in terms of OS (log-rank *p* = 0.451), CCS (log-rank *p* = 0.476), DFS (log-rank *p* = 0.678), and MFS (log-rank *p* = 0.226) (Figure 2).

## 4. Discussion

Nowadays, indications of nephron-sparing surgery are constantly increasing as a result of early diagnoses of renal masses and improved surgical quality. Therefore, recent evidence supports the indication to perform NSS whenever technically feasible [7]. Indeed, one of the most important benefits of a nephron-sparing approach is the maximal preservation of postoperative renal function [28].

Currently, the robotic approach is widely increasing, particularly in the urologic scenario, due to potential advantages in terms of intra- and postoperative complications and blood loss. Nevertheless, the advantages of robotic surgery seem to be even more evident in the setting of PN [28]. The implementation of RAPN, as opposed to the laparoscopic approach, has enabled surgeons to meet the standards set by OPN, even in the surgical management of complex renal masses with high nephrometry scores [29,30,31]. Within the setting of the NSS, renal function seems to be related to the type of resection performed (enucleation vs. enucleoresection), ischemia time, and renorrhaphy technique [32]. Recently, evidence reported that both the enucleation technique and ischemia time independently predict the occurrence of postoperative acute renal failure [26]. Acknowledging the significance of ischemia time in PN, efforts have been made to explore interventions that minimize hypoperfusion. Preoperative tumor embolization [14], super-selective clamping [33], early unclamping [34], and off-clamp PN [35] were developed to maximally preserve renal function after surgery.

Studies comparing various clamping or no-clamping techniques based on the characteristics of the tumor are lacking in the literature. In our opinion, these studies should be implemented in order to tailor the best technique according to the pre- and intraoperative features of each renal tumor. A recent PSM analysis showed that patients who underwent off-clamp PN had a higher likelihood of maintaining an unaltered eGFR compared to those who underwent on-clamp PN (58% vs. 4%, *p* 0.02). Additionally, this analysis reported a lower probability of an eGFR decline >25% in the off-C RAPN group (9% vs. 47%, *p* 0.02) [36]. Conversely, a multicenter RCT, on- vs. off-clamp RAPN, reported no significant differences in long-term functional outcomes [12], reigniting the debate about the hypothetical advantage of a purely off-clamp approach.

In this context, we reported the results of our multicenter study, where we analyzed the oncologic, functional, and perioperative outcomes of OPN and RAPN in a purely off-clamp scenario.

The off-C RAPN demonstrated superior performance compared to the open approach in various significant perioperative outcomes, including postoperative complications rate, LoS, postoperative transfusions rate, and 1 year trifecta achievement.

In a recent study, Brassetti et al. proposed a reinterpretation of the classic trifecta, substituting warm ischemia time (WIT) with the presence/absence of a significant deterioration of eGFR. This modification allows the trifecta to be extended to off-clamp procedures [26]. Additionally, this novel trifecta exhibited a better performance compared to the “Margin, Ischemia, and Complications” (MIC) score, demonstrating superiority in predicting overall survival and the risk of developing end-stage renal disease [37].

In our investigation, the achievement of the trifecta was observed in 89.8% of patients within the off-C RAPN cohort and in 80.3% of patients within the off-C OPN cohort (*p* = 0.03). Moreover, in the univariable analysis, the surgical approach emerged as the only predictor of trifecta achievement. However, it should also be mentioned that no significant differences between the two cohorts were found for CKD stage migration ≥3a (10.9% vs. 14.2%; *p* = 0.48).

In agreement with results observed in comparative studies conducted under on-clamp conditions [38,39], the rate of postoperative transfusions was lower in the off-C RAPN group (3.4% vs. 12.2%, *p* = 0.008). These results may be related to the increased abdominal pressure due to pneumoperitoneum and the better intraoperative visibility of the robot system, facilitating better management of major and minor bleeding during robot-assisted surgery [40].

In relation to the LoS, off-C RAPN demonstrated superiority over OPN (3.4 vs. 5.4, *p* < 0.001), with 64% of off-C RAPN patients experiencing a LoS within three days post-surgery, in contrast to only the 10.9% in the off-C OPN group. Moreover, these findings align with the existing on-clamp literature [38].

According to evidence already reported in the current literature, the RAPN group showed a lower rate of postoperative complications (3.2% vs. 21.7%, *p* < 0.001), particularly for low-grade complications, even if no differences were detected intraoperatively [39,40,41].

Reduced LoS and fewer postoperative complications could translate into lower hospitalization costs, offsetting the high costs associated with robotic instrumentation, particularly in high-volume centers, although further investigation is needed to be able to estimate such cost balancing [42,43].

Survival outcomes are indeed the major outcome of any genitourinary cancer treatment. A comparative study has reported that patients undergoing PN for cT2 tumors have better overall survival than those treated with radical nephrectomy [44]. In our series, positive surgical margin rates were 0% in the RAPN group versus 6.1% in the OPN group (*p* = 0.003). However, no differences were detected in terms of survival outcomes expressed as OS, CSS, DFS, and MFS, confirming the oncological safety of the robotic approach.

The field of robotic urology is on the edge of a transformative era, thanks to the synergistic integration of advanced robotics [45], artificial intelligence (AI) [46], and cutting-edge pre- and intraoperative imaging technologies [47,48]. AI algorithms are able to analyze patient data, medical history, and imaging to predict the potential for intra- and postoperative complications [49]. The successful implementation of these multifaceted technologies relied heavily on the capabilities of the robotic platform. Unlike traditional laparoscopic or open surgery, the robotic system acts as a sophisticated integration hub. It enables surgeons to fluently utilize various technologies throughout the operation. This enables surgeons to personalize surgical strategies and training [50]; therefore, it is crucial to provide accurate and up-to-date data on the various surgical strategies for partial nephrectomy in order to feed quality data into the AI models, which are essential for the algorithm to generate effective strategies tailored to each individual case.

One of the most attractive technologies in this field is the 3D virtual models (3DVMs) that, thanks to the aforementioned integration with robotic platforms, can perform augmented reality (AR) procedures driven by the superimposition of the 3DVMs [51]. The creation of the 3DVMs is the first crucial step for this kind of image-guided surgery, and additional efforts are being made to obtain high-definition models that strictly reproduce the surgical anatomy and can have a real benefit during surgical procedures [52,53]. Another potential integrable tool is intraoperative elastography [54], an emerging technology that is showing promise for improving renal tumor surgery. This technique provides surgeons with real-time information on tissue stiffness, which can be used to identify small and complex renal tumors and to guide partial kidney resection as elastography can help surgeons preserve healthy renal tissue during the resection of a tumor by recognizing diseased tissue from tumor tissue based on elastomeric characteristics [55,56]. Implementing such technology in the robotic platform could improve the quality of recorded elastomeric data and would help the robotic surgeon during PN.

We believe that all these recent technologies will be implemented in robotic surgery and enable urologists to perform safe and precision surgery tailored to the patient [57,58].

The present study is not devoid of limitations. First, even if data were collected prospectively, this study was performed retrospectively. Secondly, all robot-assisted NPs were performed in the same center, while open NPs were performed in both centers. In addition, both surgical procedures were performed by two expert surgeons in the field of renal surgery; therefore, the results obtained cannot be widely generalized.

## 5. Conclusions

In a pure off-clamp PN scenario, we confirmed the safety and feasibility of the robotic approach compared to the standard open approach. Particularly, confirming the oncological safety, we described the benefits of RAPN in terms of postoperative intercourse, supported by lower transfusion rates and length of hospital stay. Moreover, RAPN seems to provide higher surgical quality, which is expressed as a higher 1 year trifecta achievement. While these results are promising, RCT is awaited to finally establish differences between OPN and RAPN and to provide definitive conclusions regarding the superiority of one approach over the other.

## Figures and Tables

**Table 1:** Baseline features of the entire cohort and after propensity score match analysis.

Variables N (%) Mean (±SD)	Overall (542)	*Overall Cohort*		*p Value*	*PSM Cohort*		*p Value*
		*RAPN* *(395)*	*OPN* *(147)*		*RAPN* *(147)*	*OPN* *(147)*	
**Male Sex**	331 (61.1)	244 (61.8)	87 (59.2)	0.6	88 (59.9)	87 (59.2)	1
**Age (yrs)**	61.3 (±11.9)	60.2 (±12)	64.5 (±10.8)	**<0.001**	63.3 (±11.5)	64.5 (±10.8)	0.37
**BMI (Kg/m^2^)**	26.9 (±4,8)	26.8 (±5)	26.9 (±4.2)	0.87	26.9 (±5.2)	26.9 (±4.2)	0.91
**Smoking History**				**0.03**			0.15
Current	107 (19.7)	83 (21)	24 (16.3)		22 (15)	24 (16.3)	
Former	154 (28.4)	100 (25.3)	54 (36.7)		40 (27.2)	54 (36.7)	
Never	281 (51.8)	212 (53.7)	69 (46.9)		85 (57.8)	69 (46.9)	
**Diabetes**	67 (12.4)	49 (12.4)	18 (12.2)	1	22 (15)	18 (12.2)	0.6
**Hypertension**	280 (51.7)	200 (50.6)	80 (54.4)	0.4	78 (53.1)	80 (54.4)	0.9
**Tumor Side**				0.2			0.7
Right	273 (50.4)	193 (48.9)	80 (54.4)		76 (51.7)	80 (54.4)	
Left	263 (48.5)	196 (49.6)	67 (45.6)		71 (48.3)	67 (45.6)	
**Tumor Size (cm)**	4.2 (±2.3)	4.5 (±2.4)	3.5 (±1.9)	**<0.001**	3.3 (±1.5)	3.5 (±1.9)	0.47
**Renal Score**				**<0.001**			0.82
Low Risk (4–6)	172 (31.7)	103 (26.1)	69 (46.9)		72 (49)	69 (46.9)	
Moderate Risk (7–9)	244 (45)	181 (45.8)	63 (42.9)		58 (39.5)	63 (42.9)	
High Risk (10–12)	126 (23.2)	111 (28.1)	15 (10.2)		17 (11.6)	15 (10.2)	
**ASA Score**				0.5			0.16
1	75 (13.8)	53 (13.4)	22 (15)		15 (10.2)	22 (15)	
2	373 (68.8)	269 (68.1)	104 (70.7)		100 (68)	104 (70.7)	
3	94 (17.3)	73 (18.5)	21 (14.3)		32 (21.8)	21 (14.3)	
**Preoperative HB (g/dL)**	13.3 (±6.1)	14.5 (±7.1)	13.6 (±1.4)	0.1	13.7 (±1.6)	13.6 (±1.4)	0.51
**Preoperative eGFR (mL/min/1.73 m^2^)**	65.6 (±20.2)	65.6 (±19.7)	65.7 (±21.8)	0.9	64.4 (±18.5)	65.7 (±21.8)	0.58
**Preop CKD Stage**				0.76			0.81
**1**	77 (14.2)	51 (12.9)	26 (17.7)		15 (10.2)	26 (17.7)	
**2**	232 (42.8)	181 (45.8)	51 (34.7)		69 (46.9)	51 (34.7)	
**3a**	158 (29.1)	110 (27.8)	48 (32.6)		43 (29.2)	48 (32.6)	
**3b**	59 (10.9)	41 (10.4)	18 (12.2)		16 (10.9)	18 (12.2)	
**4**	16 (2.9)	12 (3)	4 (2.7)		4 (2.7)	4 (2.7)	

**Table 2:** Perioperative, functional, and pathologic outcomes.

Variables N (%) Mean (±SD)	*PSM Cohort*		*p Value*
	*RAPN* *(147)*	*OPN* *(147)*	
**Length of Stay (days)**	3.4 (±1.7)	5.4 (±1.9)	<0.001
**Intraoperative Transfusions**	1 (0.7)	5 (3.4)	0.21
**Postoperative Transfusions**	5 (3.4)	18 (12.2)	0.008
**Hb at Discharge (g/dL)**	13.1 (±11.6)	11.7 (1.6)	0.16
**Intraoperative Complications**	13 (8.8)	15 (10.2)	0.7
**Clavien–Dindo ≤ 2**	12 (8.2)	14 (9.6)	0.84
**Clavien–Dindo ≥ 3**	1 (0.6)	1 (0.6)	0.47
**Postoperative Complications**	5 (3.4)	32 (21.7)	<0.001
**Clavien–Dindo ≤ 2**	2 (1.3)	27 (18.3)	<0.001
**Clavien–Dindo ≥ 3**	3 (2.1)	5 (3.4)	0.72
**Trifecta Achievement**	132 (89.8)	118 (80.3)	0.03
**eGFR Reduction < 30%**	137 (93)	129 (88)	0.16
**Negative Surgical Margins**	147 (100)	138 (94)	0.003
**Clavien–Dindo Score < 3**	143 (97)	141 (97)	0.74
**CKD Stage Migration ≥ 3a**	16 (10.9)	21 (14.2)	0.48
**pT Stage**			
**1a**	109 (74.1)	108 (73.5)	0.9
**1b**	34 (23.1)	35 (23.8)	0.9
**2a**	4 (2.7)	4 (2.7)	1
**2b**	0 (0)	0 (0)	
**Histology**			
**RCC**	92 (62.5)	81 (55.1)	0.23
**Papillary 1**	9 (6.1)	11 (7.5)	0.81
**Papillary 2**	8 (5.4)	9 (6.1)	1
**Benign**	38 (25.8)	47 (32)	0.3

**Table 3:** Univariable analysis, predictors of trifecta achievement.

	Univariable Analysis			
	Odds Ratio	95% CI		*p* Value
		Lower	Higher	
Age	0.97	0.95	1.00	0.11
BMI	1.05	0.97	1.13	0.24
Approach	2.16	1.10	4.23	0.02
Tumor Size	0.92	0.78	1.09	0.34
Renal Score	0.99	0.83	1.17	0.92
Preop_HB	1.22	1.00	1.50	0.48
Preop_eGFR	0.98	0.97	1.00	0.10
ASA Score	0.87	0.48	1.55	0.63

## Data Availability

The data will be provided by authors under reasonable request.
